# Immunometabolic response in Egyptian water buffalo cows during the transition period

**DOI:** 10.14202/vetworld.2021.2678-2685

**Published:** 2021-10-21

**Authors:** Naglaa A. Gomaa, Samy A. Darwish, Mahmoud A. Aly

**Affiliations:** 1 Department of Animal Medicine, Faculty of Veterinary Medicine, Kafrelsheikh University, Kafrelsheikh, 33516, Egypt; 2 Mehallat Mousa Buffalo Research Station, Animal Production Research Institute, Ministry of Agriculture, Egypt; 3 Department of Animal Medicine and Infectious Disease, Faculty of Veterinary Medicine, Sadat-City University, Egypt.

**Keywords:** Egyptian water buffalo, immunometabolic changes, transition period

## Abstract

**Background and Aim:**

The transition period is extremely critical for pregnant producing animals. However, there is very limited research on the metabolic and immunological changes in Egyptian water buffalo cows during the transition period. Therefore, this study was conducted to investigate the immunometabolic changes occurring during the transition period in Egyptian water buffalo cows.

**Materials and Methods:**

A total of 50 multiparous pregnant Egyptian water buffalo cows were subjected to weekly blood sampling 3 weeks before calving and 3 weeks after calving and on the day of parturition to determine the complete blood count, including red blood cell count, total leukocyte count (TLC), differential leukocyte count, hemoglobin level, and packed cell volume (PCV). Some selected serum biochemical and immunological parameters were analyzed, including serum glucose, beta-hydroxybutyric acid (BHBA), non-esterified fatty acids, triglycerides, high-density lipoprotein, low-density lipoprotein (LDL), very LDL (VLDL), cholesterol, total protein, albumin, globulin, creatinine, blood urea nitrogen (BUN), aspartate aminotransferase, alkaline phosphatase, alanine transaminase, gamma-glutamyl transferase, Haptoglobin, and C-reactive protein and the pro-inflammatory cytokines interleukin β1, interleukin 6 (IL-6), and tumor necrosis factor-alpha. All data were statistically analyzed using the IBM Statistical Package for the Social Sciences statistics software.

**Results:**

The neutrophil count showed a statistically significant increase at 2 weeks preparturition. There was also a significant increase in PCV, TLC, neutrophil count, and IL-6 and TNF-a level at the time of parturition and even at 2 weeks post parturition, except PCV that returned to normal levels in the 1^st^ week post parturition. BHBA and BUN levels were increased significantly in the 2^nd^ and 3^rd^ weeks postcalving. Serum creatinine and VLDL levels were decreased significantly at the time of parturition, and VLDL levels showed a significant decrease even till the 3^rd^ week postcalving, whereas creatinine levels gradually returned to the pre-calving levels in the 3^rd^ week postcalving. Other parameters showed no significant changes.

**Conclusion:**

The most important immunometabolic changes occur in the first 2 weeks post parturition in Egyptian water buffalo cows, which exhibit a potent, remarkable physiological adaptation achieved by their functional liver, which can help the animal overcome the stressful conditions during the transition period.

## Introduction

The water buffalo (*Bubalus bubalis*) is a livestock species of economic significance in several countries of Asia and the Mediterranean. Egyptian water buffaloes are a major source of high-quality meat and milk in Egypt, although they breed under drastic conditions [1,2]. Water buffaloes may exceed the ability of cattle to adapt to stressful conditions, due to which metabolic disorders are less frequent in them than in cattle [3,4]. During the transition period from late pregnancy to early lactation, most dairy cattle experience a period of negative energy balance (NEB), insulin resistance, hypocalcemia, reduced immune function, and infectious diseases [5,6]. Innate immunity plays a critical role in the health of dairy cattle during the transition period [7,8]. During this period, high levels of non-esterified fatty acids (NEFA) are associated with altered innate and adaptive immune functions [9]. Innate immunity in dairy cattle is considered to be the primary defense against metabolic challenges during the transition [10,11]. The primary components of innate immunity include cellular components, predominantly white blood cells, and inflammatory response components that include pro-inflammatory cytokines and acute-phase proteins (APPs). Pro-inflammatory cytokines such as tumor necrosis factor-alpha (TNF-α), interleukin β1 (IL-β1), and interleukin 6 (IL-6) act on endocrine glands to influence the release of insulin, glucagon, glucocorticoids, growth hormone, and thyroxine [12,13]. Direct action of cytokines and alterations in circulating hormone levels of the host lead to altered patterns of energy and protein metabolism characteristic of the immune response [14,15]. Periparturient NEB has been implicated in contributing to immunosuppression. Moreover, the metabolic stress of lactation exacerbates periparturient immunosuppression [16].

Other study that investigated individual metabolic components associated with NEB have concluded that hypoglycemia alone may not exacerbate periparturient immunosuppression [17], and hyperketonemia may have multiple negative effects on aspects of immune function [18]. Ketosis may elevate the risk of mastitis in periparturient immunosuppressed cattle because several immune cell types are negatively influenced by metabolite levels, such as low concentrations of glucose and high concentrations of ketone bodies and NEFA [19]. In addition, dairy buffaloes have been reported to be very sensitive to ketosis and hypocalcemia during early lactation, whereas hypophosphatemia is more common during dry periods [20]. The physiological adaptation of metabolic processes in periparturient cows begins well before parturition and is primarily regulated by cytokines, APPs, and energetic metabolites.

At present, there is limited information regarding the alterations related to innate immunity reactants or carbohydrate and lipid metabolic profiles in the blood of dairy water buffalo cows in the transition period before the appearance of clinical signs. Therefore, this study was conducted to examine the alterations in these metabolites and the response of pro-inflammatory cytokines to these changes in Egyptian dairy water buffaloes.

## Materials and Methods

### Ethical approval

This study was approved by the Institutional Animal Ethics Committee, Faculty of Veterinary Medicine, Kafrelsheikh University, (Approval No. KFS-IACUC/8/9/2018).

### Study period and location

The study was conducted from October 2018 to December 2018. The study was conducted at Animal Production Section of Mehlet Moussa Experimental Station at the Research Institute in Kafre El-Sheikh Governorate, Egypt.

### Study design

A total of 50 pregnant, multiparous buffalo cows in the transitional period (3 weeks preparturition till 3 weeks post parturition) were included in this study. They were aged 3-14 years and had a body weight of 450-590 kg. The animals belonged to the animal production section of Mehlet Moussa Experimental Station at the Research Institute inKafr El Shiekh Governorate, Egypt. The animals were fed on a total mixed ration according to NRC guidelines (i.e., grass hay, corn silage, berseem, and commercial concentrates), and fresh, clean drinking water was supplied *ad libitum*. All animals were subjected to clinical examination, which included evaluation of rectal temperature, pulse rate, and ruminal contraction as described previously [21], and average body condition scores (BCS) were recorded based on a 5-point scale system (1=thin and 5=fat) as described elsewhere [22]. The animals selected in this study were in good health and nutritional conditions and were confirmed to be free from any external, internal, and blood parasites.

### Blood biochemical analysis

Venous blood samples were collected from each animal 7 times periodically, that is, at 3 weeks, 2 weeks, and 1-week periparturient and at calving time (from 12 to 24 h after parturition) and at 1, 2, and 3 weeks post parturient. Venous blood samples were collected from the jugular vein into two tubes as follows: 5 mL of blood in a tube without anticoagulant for serum collection and another 5 mL of blood in a tube with EDTA for hematological examination. Sera were collected by centrifugation of blood samples at 3000 rpm for 10 min, and they were stored at −20°C until analysis. Complete blood count, including red blood cell (RBC) count and total leukocyte count (TLC), was examined using a standard hemocytometer (with improved Neubauer ruling). Hemoglobin (Hb) concentration and packed cell volume (PCV) were measured using cyanmethemoglobin and microhematocrit methods, respectively. Blood smears were prepared immediately after vein puncture, and differential leukocyte counts, including neutrophils, lymphocytes, monocytes, and eosinophils, were evaluated from Giemsa-stained smears. Selected biochemical parameters such as glucose were determined by an enzymatic colorimetric method usingBio-diagnostic kits (Bio-diagnostic Company, Egypt). NEFA level was measured using diagnostic kits on photometric systems (DIA Lab, Austria). Beta-hydroxybutyric acid (BHBA) level was determined using diagnostic kits (POINTE Scientific INC, USA). The levels of triglycerides (TG), cholesterol, high-density lipoprotein (HDL), low-density lipoprotein (LDL), very LDL (VLDL), total protein (TP), and albumin (Alb) were measured spectrophotometrically using commercial test kits (Biomed Diagnostics, Germany). Plasma globulin (GLOB) levels were calculated by subtracting Alb level from the TP level, and the levels of blood urea nitrogen (BUN), creatinine, aspartate aminotransferase (AST), alanine transaminase (ALT), and alkaline phosphatase (AP) activity were measured using enzymatic colorimetric assay kits (Spectrum Diagnostics, Cairo, Egypt). The levels of pro-inflammatory mediators, including IL-β1, IL-6, and TNF-a were determined from undiluted serum samples using commercially available enzyme-linked immunosorbent assay (ELISA) kits (BioSource International, California, USA). The plates were read at 450 nm on a computerized automated microplate ELISA reader (Bio TEC, ELX800G, USA). All measurements were made in duplicate. APPs such as haptoglobin (Hp) were determined using Cobas e 411 analyzers (Germany) using Hp test kits with highly sensitive two-site ELISA. C-reactive protein (CRP) levels were also determined using a quantitative immunoturbidimetric method according to the kit’s instructions (Wiener Lab, Argentina).

### Statistical analysis

Data were statistically analyzed using the IBM Statistical Package for the Social Sciences statistics software for Windows, Version 25.0, Armonk, NY, IBM Corp. Data that were normally distributed were represented as mean±standard error (SE). The one-way analysis of variance was used to determine the difference between groups in general and in each week separately. Further comparisons among different time periods were performed using Duncan’s multiple comparison tests. Differences were considered to be significant at p≤0.05.

## Results

The physical examination performed at 3 weeks before parturition on the pregnant water buffaloes revealed that all the examined animals were physiologically normal, and all their body parameters (body temperature, heart rate, and ruminal contractions) were within the normal physiological range. The results of hematological, biochemical, and immunological examinations are presented as mean±SE in Tables-1-3, respectively.

**Table-1:** Hematological parameters before and after parturition and on the day of parturition at the following time points.

Variables	Weeks’ pre-parturition			At parturition	Weeks’ post-parturition		
		
	−3	−2	−1	0	1	2	3
RBCs×10^6^/µL	6.93±0.29	6.98±0.32	7.64±0.48	6.45±0.56	5.89±0.44	5.47±0.43	5.83±0.19
Hb (g/dL)	9.94±0.33	10.23±0.7	10.56±0.69	10.46±0.60	9.22±0.17	9.08±0.32	9.1±0.68
PCV%	35±0.96^b^	35±1.3^b^	36.0±1.7^b^	39.5±3.88^a^	33.1±1.70^c^	35.6±3.34^b^	38.1±1.68^b^
TLC×10^3^/mm^3^	8.22±0.35^b^	8.02±0.30^b^	8.30±0.25^b^	10.02±0.89^a^	10.16±0.66^a^	10.19±0.39^a^	9.71±0.51^b^
Neutrophils%	33.39±0.83^c^	36.25±0.77^b^	36.10±0.2^b^	35.12±0.85^b^	37.45±0.56^a^	37.47±0.83^a^	35.47±0.85^b^
Lymphocytes%	59.19±2.4	57.23±1.6	55.80±1.7	57.16±1.58	54.69±2.4	56.33±1.6	57.45±2.5
Monocytes%	3.72±0.76	3.53±0.55	4.78±0.45	4.09±0.65	4.08±0.35	4.04±0.44	3.63±0.77
Eosinophils%	3.70±0.2	2.99±0.34	3.50±0.56	3.45±0.55	3.78±0.43	3.16±0.22	3.45±0.44

*Data are presented as (means±standard error). *Mean values with different superscript letters in the same row are significantly different at (p≤0.05). RBCs=Red blood cells, Hb=Hemoglobin, PCV=Paced cell volume, TLC=Total leukocytic count

**Table-2:** Serum biochemical parameters before and after parturition and on the day of parturition at the following time points.

Variables	Weeks’ pre-parturition			At parturition	Weeks’ post-parturition		
		
	−3	−2	−1	0	1	2	3
Glucose (mg/dL)	56.27±2.9	55.34±0.18	55.94±0.23	56.14±0.21	53.34±0.15	54.14±0.13	55.96±0.15
BHBA (mmol/L)	1.25±0.74^b^	1.77±1.55^b^	0.93±0.46^b^	1.84±1.49^b^	1.84±0.67^b^	2.78±0.81^a^	3.09±0.47^a^
NEFA mg/dL	26.15±7.72	32.3±7.02	23.43±9.22	21.15±2.25	27.43±7.24	23.6±4.7	25.03±1.55
TG mg/dL	34.6±10.34^a^	34±6.38^a^	24.4±3.52^ab^	18±3.45^b^	15.8±1.93^b^	15.2±2.25^b^	19±3.91^b^
HDL mg/dL	30.2±3.02	33.4±3.99	28.6±1.07	37.60±8.63	39.4±3.16	38.8±6.35	33.8±3.69
LDL mg/dL	32.5±1.48	30.25±2.68	27.75±9.76	42.5±9.11	39±2.80	40.25±2.99	36±5.57
VLDL mg/dL	7.85±2.07^a^	5.55±0.66^a^	5.15±0.54^ab^	3.8±0.69^b^	3.15±0.39^b^	3.2±0.45^b^	3.3±0.78^b^
Cholesterol mg/dL	100.6±5.297	101.0±4.037	84.60±9.158	100.2±15.59	107.8±5.342	103.2±9.260	96.20±15.39
TP mg/dL	6.59±0.87	6.46±1.61	6.25±1.19	5.5±2.11	6.41±0.44	6.03±0.43	6.28±0.67
Alb mg/dL	2.86±0.67	3.16±0.55	2.98±0.62	3.05±1	3.17±0.75	3.24±0.97	3.66±0.22
GLOB mg/dL	3.73±1.51	3.29±1.21	3.32±1.59	3.49±1.59	3.49±0.86	3.24±0.49	3.05±0.52
Creatinine mg/dL	1.62±0.52^a^	1.52±0.18^a^	1.54±0.23^a^	1.23±0.25^b^	1.56±0.15^a^	1.72±0.13^a^	1.76±0.15^a^
BUN mg/dL	34±7^b^	30.48±4.32^b^	29.98±3.8^b^	28.04±3.18^bc^	36.4±2.4^ab^	37.68±2.9^a^	41.04±3.85^a^
AST IU/L	61.2±3.2^a^	55.2±0.58^b^	53.2±1.20^b^	53.4±1.60^b^	51.8±0.86^b^	56.6±0.90^b^	54±2.50^b^
AP IU/L	123.5±6.0^b^	122.7±4.0^b^	117.6±15.0^b^	129.5±19.7^ab^	100.66±9.2^b^	147.66±10.3^b^	157±12.20^a^
ALT IU/L	29±7.3 ^a^	29.4±6.6^ab^	27.4±9.1^ab^	26.56±8.8^b^	27.02±5.9^ab^	28.84±6.5^ab^	29.06±6.2^a^
GGT IU/L	16.1±2.74	14.84±2.9	14.24±41	12.3±3.5	12.4±1.14	13.2±1.6	15.5±2.59
Hp mg/dL	0.018±0.004	0.022±0.01	0.024±0.01	0.016±0.005	0.018±0.01	0.026±0.005	0.022±0.01
CRP mg/ds	6.53±0.36	6.98±0.38	7.64±0.29	6.45±0.41	5.8±0.32	5.47±0.47	5.83±0.37

*Data are presented as means±standard error. *Mean values with different superscript letters in the same row are significantly different at p*≤*0.05. ALT=Alanine transaminase, Alb=Albumin, AP=Alkaline phosphatase, AST=Aspartate aminotransferase, BUN=Blood urea nitrogen, CRP=C-reactive protein, GLOB=Globulin, GGT=Gamma-glutamyl transferase, Hb=Hemoglobin, HDL=High density lipoprotein, Hp=Haptoglobin, LDL: Low density lipoprotein, NEFA=Non-esterified fatty acids, TG=Triglycerides, TP=Total protein, VLDL=Very low-density lipoprotein, BHBA=Beta-hydroxybutyric acid

**Table-3:** Pro-inflammatory cytokines in serum before and after parturition and on the day of parturition at the following time points.

Variables	Weeks’ pre-parturition			At parturition	Weeks’ post-parturition		
		
	-1	-2	-3	0	1	2	3
IL-β1 (pg/mL)	115.3±2.5	110.9±1.8	116.6±2.4	115.5±2.1	119±1.8	114±1.7	114.1±1.9
IL-6 (ng/mL)	5.68±0.21^b^	6.38±1.66^ab^	6.66±1.57^ab^	6.88±0.2^a^	7.64±0.16^a^	6.6±0.13^a^	5.96±0.15^b^
TNF? (ng/mL)	0.58±0.03^b^	0.61±0.01^ab^	0.62±0.01^ab^	0.63±0.02^ab^	0.67±0.01^a^	0.59±0.01^b^	0.58±0.01^b^

*Data are presented as (means±standard error). *Mean values with different superscript letters in the same row are significantly different at (p≤0.05). IL-β1=Interleukin β1, IL-6=Interleukin 6, TNF-α=Tumor necrosis factor alpha

As shown in Table-1, there is a nonsignificant decrease in RBC count and Hb concentration in the 1^st^ and 2^nd^ weeks after parturition. Only PCV was significantly increased on the day of parturition. However, TLC showed significant increases at the day of calving and 2 weeks post parturition, and neutrophil count showed significant increases at 2 weeks preparturition and till the 3^rd^ week after parturition.

Table-2 shows that there was a significant increase in serum BHBA level at the 2^nd^ and 3^rd^ weeks after parturition. Serum VLDL levels began to decrease significantly on the day of parturition and 3 weeks of early lactation. Moreover, there was a significant increase in BUN levels at the 2^nd^ and 3^rd^ weeks of parturition, whereas serum creatinine levels showed a gradual decrease that became significant at the time of parturition, but the levels gradually returned to precalving levels in the 3^rd^ week after parturition. However, no significant changes were detected in the levels of serum glucose, NEFA, cholesterol, TG, HDL, LDL, serum TP, Alb, and GLOB before and after parturition. The activity of hepatic enzymes showed some fluctuation during the transition period, except serum gamma-glutamyl transferase activity that showed no significant changes during this period. The levels of serum AST, ALT, and AP activity decreased significantly at 2 weeks postpartum (Table-2). The levels of serum Hp and serum CRP also showed no significant changes during the transition period in the water buffalo cows (Table-2).

Regarding the pro-inflammatory cytokines, the levels of IL-β1 showed no significant changes among the different groups, whereas those of IL-6 were increased on the day of calving and at the 1^st^ and 2^nd^ weeks post parturition. The levels of TNF-a also showed a significant increase at the 1^st^ week post parturition (Table-3).

## Discussion

The transition period is critical in the life of dairy animals as it influences the health of dairy cattle and buffaloes and exposes them to several metabolic and inflammatory diseases [23]. All metabolic pathways are directed toward the maintenance of fetal growth or milk secretion, which is accompanied by hematological and biochemical alterations in the blood [24]. Physiologically, at the end of pregnancy, the RBC count, TLC, and platelet count increase due to the erythropoietic effect of chronic placental somatotropin, progesterone, and prolactin [25]. However, in the present study, a nonsignificant decrease in RBC count and Hb concentration was detected in the 1^st^ week of lactation. This finding can be attributed to the nutritional condition that can alter the erythropoietic effect and the lower concentrations of blood erythrocytes [26]. Furthermore, the significant increase in TLC and neutrophil count above the normal physiological limit at prepartum and during calving in buffaloes may be associated with reduced glucocorticoid receptor expression in blood neutrophils and an increase in cortisol level, resulting in neutrophilia and leukocytosis as suggested in a previous study [27]. Subsequently, the normal decline in TLC observed in the buffaloes that did not develop diseases in the later postpartum period might be due to the migration of leukocytes toward the uterine lumen and mammary gland [28]. However, only PCV was increased at the day of calving due to the loss of body fluids from uterine placentomes [29].

Blood glucose concentration is considered to reflect the carbohydrate status of animals. Glucose is an important nutrient required for normal body function, so it is generally under tight homeostatic control [30]. In this study, the blood glucose levels remained almost similar during late pregnancy and within the 1^st^ week after parturition. This result was consistent with the previous studies on buffaloes in the transition period [31,32] but inconsistent with another study [33] that reported that the average glucose concentrations varied before calving, were significantly increased at the day of calving, and then decreased again after calving.

Body lipids are considered as the source of energy during the period of starvation or NEB. During late pregnancy, after parturition, and the initiation of lactation, the animal copes with the negative energy state by utilizing the lipid reserve through lipolysis. The major blood indicators of lipid mobilization in ruminant animals are BHBA and NEFA [34].

In this study, serum BHBA levels were significantly increased in the 2^nd^ and 3^rd^ weeks after parturition, whereas the levels of NEFA, cholesterol, or HDL showed nonsignificant changes during the transition period, consistent with previous research [35]. This result indicates a lower rate of body fat mobilization in pregnant buffaloes than that occurring in transitional dairy cows [36]. Moreover, lipolysis is aggravated in dairy cows with a genetic drive for high milk production, which is not observed in buffaloes [37]. In addition, most buffaloes included in this study had low or acceptable BCS (2.5–4) at the time of parturition, which results in a low rate of lipolysis [26]. Although lower serum NEFA levels in dairy buffaloes during the transition period were reported in several studies, the exact reason still remains poorly understood [38]. In the late stages of pregnancy, circulating NEFA is excessively taken up by the liver and esterified to triglycerides, which are then secreted into the blood in the form of VLDL. Immediately after parturition, the synthesis of hepatic triglycerides exceeds the secretion of VLDL, leading to the accumulation of triglycerides in the liver [39]. This explains the decrease in serum VLDL levels immediately after parturition in the present study. At calving and early lactation, increasing glucagon and glucocorticoid concentrations stimulate hepatic glycogen and triglyceride degradation and promote β-oxidation and ketone production [23]. This explains the increase in serum BHBA levels after parturition in this study.

Circulating proteins, particularly Alb, may be associated with the health condition of dairy cows and provide information about hepatic function during the peripartum period [23]. In the present study, no significant changes were observed in the TP, Alb, and GLOB levels, which confirm the healthy condition of the liver; this was further confirmed by the nonsignificant variations in hepatic enzyme activities during late pregnancy and early lactation. This finding isagreed with other previous research on cows [40]. However, it is consistent with another study [32] that reported that buffaloes are less sensitive than cows in developing fatty liver postpartum. However, BUN levels were significantly increased at the 2^nd^ and 3^rd^ weeks postpartum as a result of body protein catabolism after parturition due to increased protein demand for milk production, which contributes to increasing BUN levels postpartum [26]. In contrast, glomerular hyperfiltration is a typical physiological adaptation to pregnancy, reflected by a decrease in the levels of serum creatinine with advanced gestation [41]. This can be explained by the gradual decrease in serum creatinine levels preparturition that was significantly decreased at parturition and then increased gradually in the period post parturition.

APPs play a major role in the innate immune response and are primarily coordinated by cytokine-mediated production, which serve to both prevent inflammation and initiate inflammatory processes, as well as eliminate potential pathogens and contribute to healing [42]. The most important APPs in ruminant animals are Hp and CRP that provide an early nonspecific defense mechanism against injury, infection, or inflammation before the response of specific immunity [43]. Hp is recognized as a marker of inflammation in dairy cows; however, its concentration is also found to increase in a week after calving [44]. This period is associated with NEB, which is caused by limited feed intake and higher energy requirements due to increased milk production that is also related to environmental and management changes [45]. All these factors cause an increase in nonspecific inflammatory markers in the blood depending on the degree of health complications and environmental stressors, as reported by a previous that serum CRP is a good marker of herd health status, but its concentration increases in a diseased condition more than that during stress and lactation [46]. In the present study, there was a nonsignificant increase in Hp levels in the 1^st^ week after calving, whereas CRP levels showed no significant changes during the transition period. This finding indicates that buffaloes can tolerate stressful conditions during the transition period.

Inflammation is believed to be an important event during parturition as well as during early postpartum. Occurrences of obvious inflammatory reactions without clear signs of infections or any pathological conditions have been reported during the transition period [47]. Mastitis, metritis, and retained placenta are the three common diseases linked to a compromised immune system postcalving [48]. Several mechanisms that can cause immunosuppression have been suspected at the time of parturition. It has been reported that the metabolic changes during the transition period in cattle are important determinants of immunosuppression, especially increased levels of BHBA and NEFA that are associated with phagocytosis and increased oxidative activities and necrosis [49].

IL-1β, which is secreted from the placenta, directly upregulates the expression of tumor necrosis factor-α (TNF-α) in the liver. These cytokines and APPs have negative effects on the satiety center of the brain, which results in hypoinsulinemia, hypoglycemia, and depressed feed intake, leading to NEB and consequent increase of adipose tissue lipolysis. Consequently, in the present study, there was a nonsignificant increase in the levels of pro-inflammatory cytokines in the prepartum period as a result of NEB and lipolysis. At the time of parturition and postcalving, there was a rapid decrease in the levels of pro-inflammatory cytokines, especially IL-6 and TNF-α, which is consistent with previous research on cattle [50]. Meanwhile, macrophages in the adipose tissue secrete cytokines and other pro-inflammatory mediators into the liver, which causes metabolic inflammation through the upregulation of IL-6 levels. Excessive NEFA, cytokine, and BHBA accumulation in the liver reduces energy production and metabolism. IL-6 plays a central role in a wide range of liver-specific functions in cattle, such as lipoprotein metabolism, fatty acid oxidation, urea cycle, oxidative stress, transcription regulation, and protein degradation through proteasomes. A recent study indicated that hepatic inflammatory responses during the periparturient period are an important driving force for fat mobilization [51].

IL-6 and TNF-α are cytokines produced by T cells. Consequently, a significant increase in IL-6 levels was observed at calving and in the first 2 weeks postcalving. However, TNF-α level was significantly increased only during the 1^st^ week after calving. This result was consistent with another study that reported the presence of hematological inflammatory reactions without signs of infection or pathological conditions during the transition period; this was related to a brief spike in inflammatory signals to physiological adaptations to lactation and helped at the end of pregnancy [47]. Nevertheless, failure to rapidly resolve these inflammatory reactions or signals may lead to an adverse impact on the productivity, health, and fertility of the animal. Furthermore, their lower expression in the endometrium immediately after calving impairs the chemotaxis and activation of neutrophils and results in the development of endometritis in cows [42]. Inflammation is believed to be an important event during parturition as well as during early postpartum. Therefore, the pattern of inflammation during the transition period, particularly during early lactation, is a deciding factor of long-term outcomes.

## Conclusion

The most critical immunometabolic changes occur in the 1^st^ and 2^nd^ weeks postpartum in water buffalo cows. Monitoring these immunometabolic changes is extremely important for the detection of any subclinical metabolic or fertility disorders that could have a negative impact on the production and reproduction of buffalo cows. Under normal nutritional conditions, Egyptian water buffalo cows have a potent, remarkable physiological adaptive mechanism that is achieved by their functional liver and immune system, which can help them overcome the stressful conditions during the transition period.

## Authors’ Contributions

NAG and SAD: Contributed to the conception and design of the study. NAG: Wrote this manuscript. NAG, SAD and MAA: Contributed to data collection. NAG and MAA: Participated in laboratory analysis of the samples. NAG and MAA: Performed analytical work. NAG, SAD and MAA: Critically revised the manuscript. All authors read and approved the final manuscript.
